# Effects of Caffeine Exposure on Behaviour, Development and Physiology of the Freshwater Snail *Physella acuta* (Draparnaud, 1805)

**DOI:** 10.3390/toxics14010014

**Published:** 2025-12-22

**Authors:** Ahlam Mohamed-Benhamu

**Affiliations:** Grupo de Bioinformática y Ecotoxicología Molecular de Invertebrados, Facultad de Ciencias, Universidad Nacional de Educación a Distancia (UNED), Av. Esparta S/N, 28232 Madrid, Spain; ahlam.mohamed@ccia.uned.es; Tel.: +34-91-398-7644

**Keywords:** Gastropoda, freshwater, behaviour, embryonic development, sub-lethal effects, psychoactive substance

## Abstract

Caffeine (CAF), a prevalent psychoactive compound, has been identified as a significant environmental pollutant in freshwater ecosystems. This study investigates the behavioral and physiological effects of CAF at environmentally relevant concentrations (0, 5, 30, and 50 µg/L) on the freshwater snail *Physella acuta*, with a focus on both adult and embryonic responses. Adult snails were evaluated for alterations in speed, exploration, overall activity levels, and feeding behaviors, while embryos were assessed for heart rate and developmental changes. The study encompassed both short-term (24 h) and mid-term (7 days) exposure periods. Low CAF concentrations (5 and 30 µg/L) were found to enhance adult movement and exploratory behavior in the short term, whereas prolonged exposure resulted in a decline in these behaviors. A high CAF concentration (50 µg/L) consistently diminished movement and feeding in adults. Embryos exhibited a dose-dependent increase in heart rate and manifested malformations at elevated concentrations. These findings provide insights into the impact of CAF on freshwater invertebrates and contribute valuable data for ecological risk assessment.

## 1. Introduction

Caffeine (1,3,7-trimethylxanthine, CAF) is recognized as an emerging pollutant in aquatic ecosystems [1,2,3,4]. Classified as a psychoactive substance and central nervous system stimulant from the methylxanthine group [5] is widely consumed due to its stimulating effects. The primary source of CAF in the environment is attributable to anthropogenic activities, as a constituent of numerous widely consumed products, including coffee, tea, chocolate, pharmaceuticals, and the increasingly popular category of energy drinks [6]. CAF can enter aquatic ecosystems through the discharge of wastewater from residential areas, industrial sites, and coffee processing facilities [7,8]. While CAF confers certain beneficial effects on humans, its extensive utilization has resulted in its emergence as one of the most ubiquitous pharmaceutical and personal care products (PPCPs) in the environment [9], thereby serving as an indicator of pollution [6]. These characteristics of CAF result in its concentration within environmental matrices surpassing the rate of degradation [4,10,11].

Beyond its well-known effects on humans, CAF also influences a wide range of aquatic organisms [1]. It has been shown to induce cell death, alter locomotor behavior, and interfere with cell cycle regulation in both target and non-target species, including invertebrates and vertebrates [1]. In aquatic organisms, CAF has been shown to exert adverse effects even at environmentally relevant concentrations. These effects include oxidative stress, lipid peroxidation, neurotoxicity, mutagenic activity, alterations in energy reserves and metabolic function, impaired reproduction and development, and, in some cases, increased mortality [12,13].

The toxicity of CAF in aquatic environments is largely due to its high water solubility and low log Kow value, which facilitate its absorption through biological membranes [10,11]. Despite its high aqueous solubility, CAF readily crosses biological membranes due to its small molecular size, neutral charge at physiological pH, and moderate lipophilicity. This enables CAF to penetrate cells and tissues with high specificity and efficiency, amplifying its biological effects [7]. CAF is considered relatively stable in aquatic environments, with reported half-life estimates ranging from approximately 100 to 240 days, and up to several years under constant low-temperature conditions (8–20 °C) [14]. However, these estimates are largely derived from abiotic degradation kinetics under simplified conditions and do not fully account for biological degradation, dilution, or other environmental loss processes. Consequently, CAF persistence in aquatic systems is highly context-dependent and may be overestimated when extrapolated to biologically active or environmentally complex systems.

CAF is frequently detected in a wide-range of water bodies at varying concentrations. In wastewater influents and effluents, levels have been reported to range widely from 0.02 to 86,000 µg/L, while in surface waters such as lakes and rivers, concentrations typically range from 0.05 to 33.2 µg/L. Groundwater concentrations occasionally reach up to 0.68 µg/L, and drinking water shows levels from 0.50 to 35 µg/L. Reservoirs have recorded concentrations as high as 27.7 µg/L [1]. According to [15], CAF in wastewater commonly ranges from 20 to 300 µg/L. Some of the highest surface water concentrations have been reported in Costa Rica, reaching 1.1 mg/L [13] and in northwestern Spain, where levels reached up to 44.6 µg/L [16].

Based on this background and primarily considering the high consumption of caffeine worldwide, it is hypothesized that although CAF is often considered a low-priority contaminant, its persistence and potential biological activity in aquatic environments may cause significant sub-lethal effects on non-target organisms [17]. Such effects have not been clearly demonstrated in freshwater gastropods, and since species as *Physella acuta* form a fundamental base of freshwater trophic chains, understanding these impacts is crucial.

In this study, the ecotoxicological impacts of CAF were evaluated using adults and embryos of *P. acuta*, a sensitive and ecologically relevant pulmonated gastropod species commonly found in freshwater ecosystems worldwide. This gastropod native to North America and introduced into Europe, plays a significant role in aquatic food webs, functioning as both a grazer and a detritivore. *P. acuta* typically has a lifespan of about one year (reduced to 4–6 months under suboptimal conditions) [18]. Its short life cycle, high reproductive rate, and rapid generational turnover enhance its usefulness in ecological and toxicological studies. Additionally, its simple and accessible nervous and circulatory systems make it a valuable model organism for toxicological research. Furthermore, as an air-breathing snail, it is particularly sensitive to variations in water quality and environmental stressors.

This study evaluates behavioural changes and feeding rates in adult snails, while also examining developmental and physiological responses in embryos, such as alterations in heart rate and developmental progression. This dual-life-stage approach offers a comprehensive assessment of the sub-lethal effects of CAF and their potential ecological implications. Given CAF’s high water solubility, environmental stability, and established potential to induce neurotoxic effects as well as reproductive and developmental impairments [10], this research aims to enhance understanding of its impact on aquatic invertebrates and the potential risks it poses to freshwater ecosystems.

## 2. Materials and Methods

### 2.1. Chemicals and Test Concentrations

Caffeine (C_8_H_10_N_4_O_2_, molecular weight 194.19 g/mol) was purchased from Sigma-Aldrich Chemical Spain (CAS number: 58–08-2), in a degree of purity of 99%.

The nominal concentrations stipulated for the exposures were based on the array of concentrations already detected in distinct environmental compartments, varying from low nanogram to intermediate micrograms per liter [15]. Following this principle, three environmentally relevant CAF concentrations were chosen for exposing the test organism *P. acuta*: 5, 30, and 50 μg/L (25.75, 154.49, and 257.48 nM).

**Figure 1:**
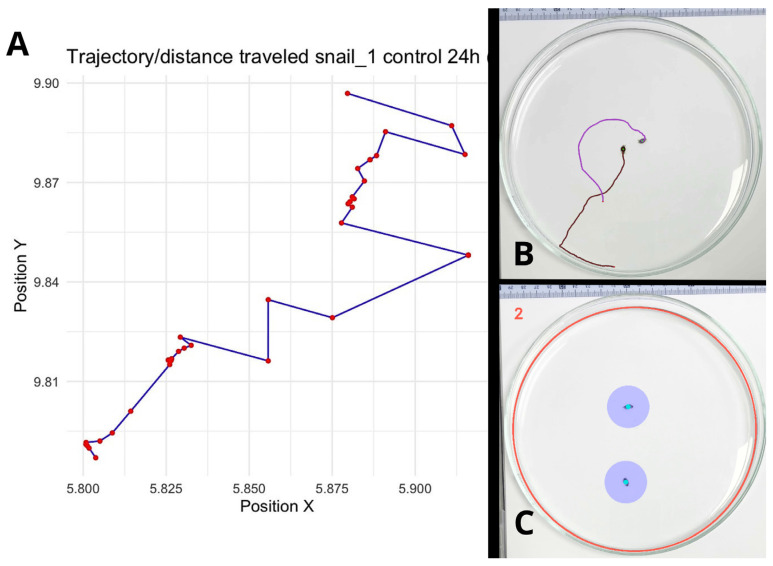
Analysis of behavioral patterns in *P. acuta* using the tracking software AnimalTA v3.0 [24]. (**A**): The analysis software refers to the parameter as “Distance travelled,” which represents the sum of all segments (trams) covered by the snail, equivalent to the trajectory (Shown here: a 2D representation of the coordinates of one control snail at 24 h, visualized using R (version 4.1.2) to illustrate the trajectory.) (**B**): Example of a trajectory map generated by the software for two control-treatment snails at 24 h. (**C**): Measurement of exploration, defined as the number of newly visited cells, pixels that fall within a circular area centered (purple) on the snail’s position as it moves. The number “2” indicates that there are two snails (measurement targets) in the arena.

A stock CAF solution (3 mg mL^−1^) was prepared in distilled water and stored at 4 °C, following [19] with the exception that it was not protected from light. NMR analysis (see Figure 1) confirmed no photodegradation over three weeks, supporting the omission of light protection and the decision not to refresh the medium during experiments. The stock remained stable for at least one month.

CAF solutions with final concentrations of 5, 30, and 50 μg L^−1^ were freshly prepared by conducting appropriate serial dilutions of the stock solution to achieve intermediate concentrations. These intermediate solutions were subsequently incorporated into the exposure medium to attain the specified final concentrations (5, 30, and 50 μg L^−1^) within a total volume of 100 mL of *P. acuta* medium for each experimental treatment. All solutions were freshly prepared prior to use and mixed to ensure homogeneity.

Additionally, CAF is known to exhibit high solubility in freshwater (approximately 20 mg/mL) at room temperature [20]. Due to its high solubility and low hydrophobicity, adsorption onto the glass surfaces of the laboratory glassware used in the experiments is not expected. This physicochemical profile supports the assumption that the measured CAF concentrations reflect actual values in the aqueous medium, without losses due to surface adsorption.

### 2.2. Test Organism and Experimental Setup

*Physella acuta* (Gastropoda, Pulmonata, Basommatophora) is a hermaphroditic species that mainly practices outcrossing. Before the experiment, the organism was maintained in a climate-controlled environment at 18 °C for several generations. The culture conditions were defined previously [21,22]. In summary, mature snails were kept in glass vessels with 250 mL of culture medium (2 mM CaCl_2_, 0.5 mM MgSO_4_, 0.77 mM NaHCO_3_, and 0.08 mM KCl) at 18 °C under a 16:8 light-dark cycle for breeding purposes. Mature snails produced egg masses containing embryos, which developed directly into juveniles in a time lapse of approximately 10 days. As the juveniles matured into adults, their first oviposition occurred approximately two months after hatching.

To evaluate CAF effects in adults (locomotion and feeding behaviour), both short-term (24 h) and mid-term (7 d) exposures were performed in adults to assess behavioural changes. The 7-day timeframe was selected as it is commonly considered sufficient to observe non-acute molecular changes, including shifts in gene expression or cellular stress responses [22,23]. Therefore, this exposure duration can be reasonably categorized sub-chronic/mid-term, especially in the context of studying mechanisms of toxicity or physiological adaptation.

Developmental and cardiac endpoints were monitored from time zero to day 14, covering the embryonic stage through juvenile hatching. The embryonic stage was chosen for heart rate measurements due to shell translucency, enabling non-invasive visualization.

Each condition was tested in three independent experiments to ensure reproducibility. Survival was monitored daily, along with signs of stress such as shell damage, avoidance, or retracted posture. No mortality or avoidance was observed at any CAF concentration. Additionally, oviposition (number of egg masses laid) was initially considered as a reproductive endpoint; no significant differences were observed between treatments during the exposure period. As such, this parameter was excluded from further analysis and discussion, as it did not provide additional insight. The lack of variation may suggest that oviposition is either less sensitive to CAF exposure or that other behavioral and physiological endpoints (e.g., heart rate, locomotion, feeding rate) are more suitable early indicators of sublethal stress in *P. acuta*.

### 2.3. Behaviour Patterns and Locomotion Experiments

Each experimental unit was a glass vessel with 100 mL of *P. acuta* medium (as previously described) added to achieve the final volume required for exposures at the specified CAF concentrations. Each treatment involved n = 5 snails. Each treatment was evaluated in three independent experimental trials in a climate-controlled environment at 18 °C, with two individual snails analyzed per treatment in each trial (n = 6), ensuring consistency and reproducibility across replicates.

Locomotor activity was assessed by measuring the average speed (cm/s), distance travelled (=trajectory, in cm), and exploration. Exploration was quantified by measuring the area around each animal’s position at each frame, assuming a circular area centered on that location (AnimalTA v3.0; [24]; Figure 1). A pixel was classified as visited if it fell within this circular area at least once during the video; otherwise, it is considered unvisited. This approach allows for the calculation of the proportion of the arena explored (Figure 1).

All behavioral assays were performed in a clean, large glass Petri dish (145 mm diameter) containing 100 mL of medium. A 2 min observation period was used, consistent with the protocol described by [25]. Prior to video recording, snails were allowed to acclimate for approximately 5 min in the observation dish used for the evaluation, in accordance with [26]. Two snails per treatment group were randomly selected for a duplicate recording session. Movement was recorded using a phone camera setup iPhone 13 smartphone (Apple Inc., Cupertino, CA, USA). All behavioral recordings were analyzed using AnimalTA v.3.0 tracking software [24], which tracked individual snail movements and computed quantitative endpoints and spatial exploration patterns (area explored scores evaluated by comparing the measured scores to those of the control group, which served as the reference standard).

### 2.4. Coordinated Movements

In addition to quantitative assessments, a qualitative observation of locomotion was considered using predefined behavioural criteria. The behaviour was defined as “Normal” when the snail showed smooth gliding, symmetrical tentacle and foot movements, and timely responses to tactile or environmental stimuli. On the other hand, it was considered “Impaired locomotion” when the snail showed erratic movement (e.g., sudden direction changes, irregular speed, spasmodic motions, repetitive circling, disorientation) or delayed withdrawal responses. Uncoordinated or atypical behaviors were documented through video analysis to aid interpretation of sublethal effects.

### 2.5. Feeding Behaviour Assays

Feeding performance was analyzed using a custom method designed to assess the impact of CAF on both spontaneous and evoked feeding behaviour. To assess feeding rate, a nutritive capsule was prepared by dissolving agar at a concentration of 3.8% (*w*/*v*) in Ultrapure water (Milli-Q) produced using a Milli-Q water purification system (MilliporeSigma, Burlington, MA, USA). A total of 5 mL of this agarose solution was prepared, corresponding to 0.19 g of agar. After complete dissolution, approximately 0.2 g (equivalent to about two small lab spatula spoons) of TetraMin^®^ tropical fish flakes were added and thoroughly mixed into the solution. The resulting mixture was transferred into a spherical mold and allowed to solidify at 4 °C, forming a soft, uniform capsule.

Individual snails were placed in glass Petri dishes (145 mm diameter) with 100 mL of *P. acuta* medium, consistent with the behavioural assay setup. Two snails per treatment were randomly selected for duplicate recording sessions (n = 6, based on three independent experiments). After food capsule introduction, the time to perceive, approach (direct movement or head contact), and initiate rasping was recorded. Recordings (1:50–2:00 min) captured behavioural parameters including food location ability (min), average rasping duration (min), and overall feeding activity. Feeding activity was scored on a 0–3 scale, developed from behavioural observations in this study, as follows: **Score (0–3): 3—High Response:** actively seeks out food; demonstrates clear behaviors indicating awareness of food location and hunger (e.g., movement toward food source, rasping, and searching behaviors; less than 10 s). **2—Moderate Response:** occasionally responds to food or hunger cues; may take longer to approach food or exhibit less consistent searching behavior (30 to 50 s). **1—Low Response:** rarely responds to hunger or food cues; minimal searching or approach behavior (more than 50 s). **0—No Response:** Completely inactive; no observable response to the presence of food or hunger cues.

### 2.6. Monitoring Embryo Development and Cardiac Activity

In a climate-controlled environment at 18 °C, 24 h period egg masses produced by *P. acuta* snails were collected and assigned to individual glass Petri dishes for each treatment, which included control and three distinct CAF concentrations (5, 30, and 50 µg/L) in a final volume of 10 mL of *P. acuta* medium across three independent experiments. The eggs contained within these egg masses were subjected to daily examination and recorded at 24-h intervals using a Nikon SMZ-2T binocular microscope equipped with a MOTIC A1 (MotiConnect ×64) camera. Observations were made regarding development and malformation in accordance with each *P. acuta* developmental stage marker (Table 1) and were scored on a standardized 0–5 scale, where 0 indicated arrested or malformed embryos, and 5 indicated full development with shell and heart formation (Table 2).

Cardiac function was evaluated by measuring heart rate in embryonic snails at 6, 7, 9, and 10 days post-fertilization, once cardiac development was complete (approximately 6 days). This assessment aimed to determine physiological alterations induced by CAF exposure and to explore its potential as a biomarker for pollutant effects in aquatic invertebrates.

Measurements were taken both before and after hatching, with embryos kept within egg masses to minimize handling stress. Heartbeats were manually counted for one minute, and the mean of two independent counts per individual was used. Concurrently, heart activity was also recorded, and irregular beats were documented. Baselines were established in controls (non-CAF-exposed) snails. CAF-exposed groups were compared to controls through statistical analysis (ANOVA or Kruskal–Wallis, depending on normality and variance homogeneity). Following [27], heartbeat rates of snails within the same dish were averaged prior to analysis.

On day 14, the number of newly hatched juveniles was recorded to assess hatching success in both control and CAF-treated groups. Hatching was defined as the emergence of juveniles from both the egg and the egg mass [28]. Hatching success was calculated as:

$$H_{s}=\frac{J_{h}}{E}100$$

where H_s_ is Hatching success, J_h_ is the number of juveniles hatched, and E is the total number of eggs in the egg mass.

Additionally, a table detailing all observed malformations is included in the Supplementary Material (Table S1 and Figures S1–S3).

### 2.7. Statistical Analysis

To evaluate the effects of CAF on various behavioural endpoints, a Kruskal–Wallis test was conducted because the residuals of the data did not meet the assumptions of normality required for parametric tests. CAF concentration was treated as the intersubject factor (0, 5, 30, and 50 µg CAF/L), while time served as the intrasubject factor (24 h and 7 days of exposure). The dependent variables included the mean number of animals locating food, the mean duration of food rasping by animals, the mean speed, the mean distance travelled, and exploration scores.

In cases where the Kruskal–Wallis test indicated significant results, a *post-hoc* analysis using the Dunn test was performed to assess differences between each CAF concentration and the control group. A *p*-value of less than 0.05 was set as the criterion for statistical significance. All statistical analyses were performed in R (version 4.1.2) using the RStudio graphical interface (Posit Software, PBC, Version 2024.04.2+764).

### 2.8. NMR Photodegradation Analysis

Prior to initiating the experimental assessments and considering previous studies demonstrating CAF’s susceptibility to light and its degradation under natural environmental conditions, with some recommending the protection of stock solutions from photodegradation using aluminium foil [29], the photodegradation of CAF was investigated.

To evaluate the potential photodegradation of CAF, a solution containing 0.55 mg of CAF in 0.6 mL of deuterated water (D_2_O) (0.092% (*w*/*v*)) was analyzed using proton NMR spectroscopy at 13 °C. Solution NMR spectra were recorded on a Bruker Avance III 400 (9.4 Tesla, 400.15 MHz for 1H and 100.62 MHz for 13C) spectrometer with a 5-mm direct-detection H-F-X probe equipped with a z-gradient coil, at 300 K. Chemical shifts (δ in ppm) are given from internal solvent, MeOD-d4 4.78 for 1H and 49.2 for 13C. Typical parameters for 1H NMR spectra were spectral width 6700 Hz and pulse width 14.0 μs at an attenuation level of −12.23 dB. Typical parameters for 13C NMR spectra were spectral width 24 kHz, pulse width 12.5 μs at an attenuation level of −6 dB and relaxation delay 2 s, WALTZ-65 was used for broadband proton decoupling; the FIDS were multiplied by an exponential weighting (lb = 1 Hz) before Fourier transformation.

**Figure 2:**
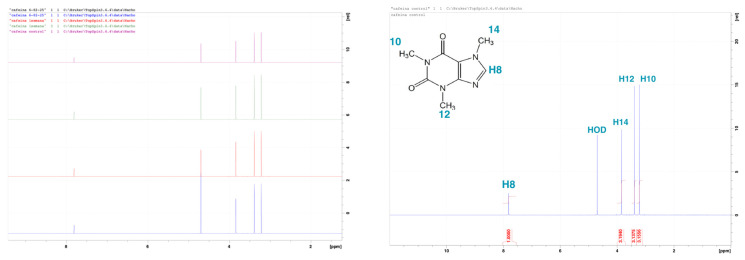
The stacked ^1^H NMR spectra of CAF methyl protons illustrate photostability, with an overlay of the control sample at t = 0 h (**right**) and a comparison of the control with other timepoints after three weeks of light exposure (**left**). The consistent chemical shifts and signal intensities suggest an absence of photodegradation. The X-axis (δ ppm) represents the chemical shift in parts per million (ppm), a standard unit in NMR, providing insights into the chemical environment of hydrogen atoms (^1^H NMR). The Y-axis shows each spectrum offset vertically, corresponding to different timepoints (in the left graph, from top to bottom; purple = control, green = 1 week, red = 1.5 weeks, and blue = 3 weeks). The spectra indicate peaks representing protons within the molecule.

The control spectrum (t = 0; Figure 2) displayed the characteristic three methyl proton peaks of the CAF molecule. Subsequent ^1^H NMR spectra were acquired after 5 days, 1 week, 2 weeks, and 3 weeks of continuous light exposure under controlled temperature conditions. As illustrated in Figure 2, the Y-axis shows each spectrum vertically offset to represent different timepoints (in the left graph, from top to bottom: purple = control, green = 1 week, red = 1.5 weeks, and blue = 3 weeks). The colored spectra display peaks corresponding to methyl protons within the molecule. Across all timepoints, the stacked spectra revealed no significant changes in peak position, shape, or intensity. The chemical structure and signal integrity remained unchanged, indicating that CAF remains chemically stable under the storage and handling conditions used in this study. This consistent signal profile confirms that the compound does not undergo photodegradation over the tested period.

The experimental findings contradict previous assumptions regarding CAF’s photodegradation [29], that recommended light protection for CAF stock solutions. Based on this NMR data, stock solutions were stored under refrigeration without light protection, as additional shielding was considered unnecessary. This stability justified the decision not to renew the exposure medium during the experimental period.

## 3. Results

### 3.1. Behaviour Assays

#### 3.1.1. Average Speed Moving

**Figure 3:**
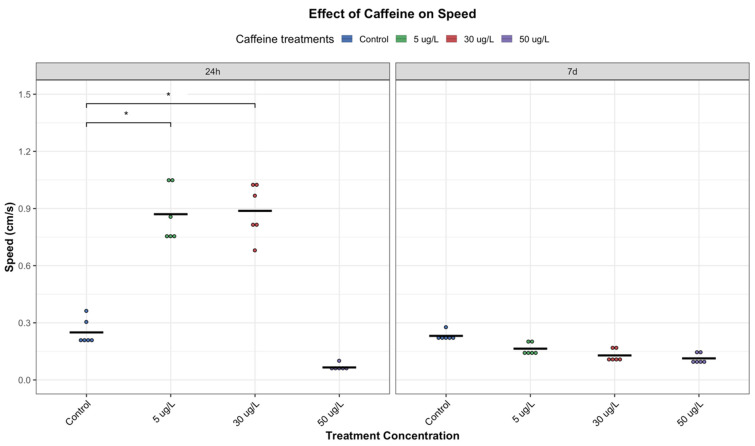
Effects of CAF on the speed of *P. acuta*. Following exposure to CAF at low (5 µg/L), medium (30 µg/L), and high (50 µg/L) concentrations, significant differences from the control group (*p* < 0.05) are indicated by asterisks. Additionally, significant differences in travelled distance between 24 h and 7 days were observed for both low and medium exposure groups. Data were visualized using RStudio (Posit Software, PBC, Version 2024.04.2+764).

Analysis of average speed revealed significant dose- and time-dependent effects of CAF on the locomotion of *P. acuta* (Figure 3). At 24 h, both low (5 µg/L) and medium (30 µg/L) concentrations of CAF resulted in increased snail speed compared to unexposed controls, suggesting an initial stimulatory effect of CAF on the species. Notably, the medium-dose group exhibited the highest average speed at this short-term exposure. However, a significant reduction in speed was observed in this group after 7 days (CAF 30 µg/L, 24 h vs. 7 days * *p* = 0.0028), indicating that the stimulatory effect may be acute and diminished over time (Figure 3).

Similarly, the low-dose group also showed a substantial decline in speed at the 7-day time point, perhaps due to physiological adaptation, metabolic elimination of CAF, or other mid-term effects. In contrast, the high-dose group (50 µg/L) did not display a time-dependent shift in response (Figure 3). At both 24 h and 7 days, snails exposed to the highest CAF concentration exhibited reduced speeds compared to the control group (not significant enough but may suggest a consistent inhibitory or toxic effect at this CAF level). Overall, by day 7, snails in both the medium- and high-dose groups displayed reduced locomotor activity, with average speeds approaching below control levels (Figure 3).

#### 3.1.2. Travelled Distance (Trajectory)

**Figure 4:**
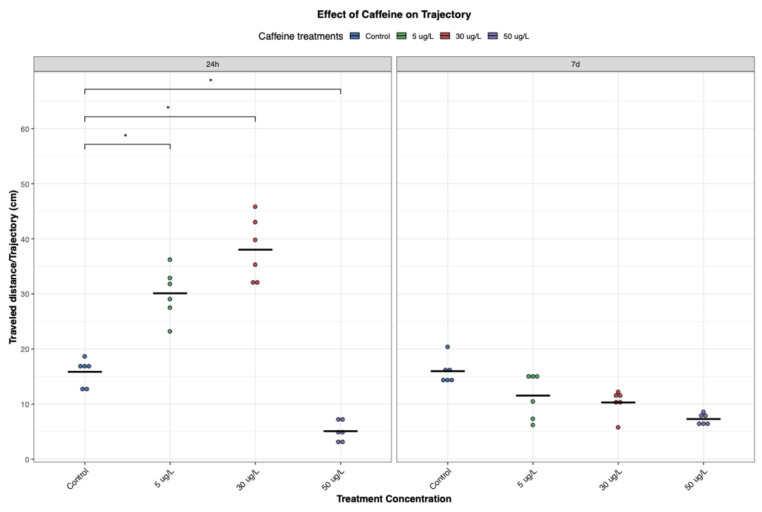
Effect of CAF on the Trajectory of *P. acuta*. Following exposure to CAF at low (5 µg/L), medium (30 µg/L), and high (50 µg/L) concentrations, significant differences from the control group (*p* < 0.05) are indicated by asterisks for these three treatments. Additionally, significant differences in travelled distance between 24 h and 7 days were observed for both low and medium exposure groups. Data were visualized using RStudio (Posit Software, PBC, Version 2024.04.2+764).

The distance travelled by animals is a key ecological variable that links behaviour, energetics, and demography, and in this study, it further highlighted the dose- and time-dependent effects of CAF on *P. acuta*. The results revealed a pattern consistent with the speed measurements (Figure 3 and Figure 4). At 24 h, snails exposed to the medium CAF concentration (30 µg/L) travelled the greatest distance, exhibiting higher trajectories and wide spatial coverage. This may suggest a stimulatory effect of CAF at this concentration in snails. This was followed by the low-dose group (5 µg/L), both indicating enhanced locomotor activity following acute CAF exposure.

Statistical analysis revealed significant differences in the distance travelled between the low-dose groups at 24 h and 7 days, as well as between the medium-dose groups at the same timepoints, indicating a clear reduction in locomotor activity over time (*p* < 0.05). This pattern mirrors the changes observed in snail speed, suggesting that the initial stimulatory effect of CAF may diminish with prolonged exposure (Figure 3 and Figure 4).

Similar time- and dose-dependent patterns have been reported previously, with acetylcholinesterase (AChE) activity implicated as a potential mechanism. The authors of [30] found that CAF induced AChE activity at low concentrations but suppressed it at higher levels. Although AChE was not measured here, this response could explain the observed behaviours: elevated AChE activity may enhance locomotion at low doses, while reduced activity at higher doses could contribute to decreased movement.

At 24 h, snails in the high-dose group (50 µg/L) travelled significantly less than controls and the lower-dose groups (*p* < 0.05), perhaps indicating early inhibitory or toxic effects (Figure 4). After 7 d, we found a significant decreasing trend across concentrations (*p* = 0.00013, Jonckheere-Terpstra test). The highest concentration consistently produced the lowest activity at both timepoints, suggesting sedative, fatigue-related, or neurotoxic effects. These findings suggest that while low to moderate doses of CAF can transiently enhance movement, sustained exposure or higher concentrations lead to suppressed locomotor behaviour, likely due to cumulative physiological stress or neural impairment.

#### 3.1.3. Exploratory Behaviour

**Figure 5:**
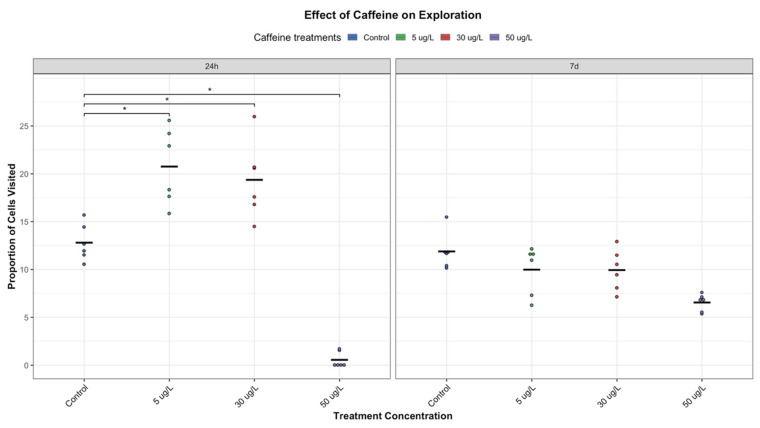
Effect of CAF on Exploration of *P. acuta*. Following exposure to CAF at low (5 µg/L), medium (30 µg/L), and high (50 µg/L) concentrations, significant differences from the control group (*p* < 0.05) are indicated by asterisks. Additionally, significant differences in exploration between 24 h and 7 days were observed for both low and medium exposure groups. Data were visualized using RStudio (Posit Software, PBC, Version 2024.04.2+764).

In *P. acuta*, exploratory behaviour is essential for both survival and reproductive success. In this study, exploration of snails was significantly enhanced for the low and medium treatments (5 µg/L and 30 µg/L of CAF, respectively) at the short-term of 24 h relative to controls (Figure 5). However, this effect was not sustained; by day 7, exploratory activity in both treatment groups was markedly reduced, showing a significant decreasing trend across concentrations (*p* = 0.00015, Jonckheere-Terpstra test), following a similar time-dependent pattern to that observed for trajectory (Figure 5).

The high-concentration group (50 µg/L) also exhibited significantly reduced exploratory behaviour at 24 h (*p* = 0.0105), indicative of possible motor suppression or, again, early signs of CAF-induced toxicity (Figure 5). These results further support a biphasic behavioural response to CAF, with acute stimulation at low to moderate doses and short-term and suppression at higher exposure. Additionally, while total distance travelled and mean speed remained largely unchanged across treatments at 7 days (Figure 3 and Figure 4), a modest increase in spatial exploration was noted at higher concentrations (Figure 5). This observation may reflect a specific modulation of exploratory behaviour, independent of general locomotor performance.

### 3.2. Feeding Behaviour

**Figure 6:**
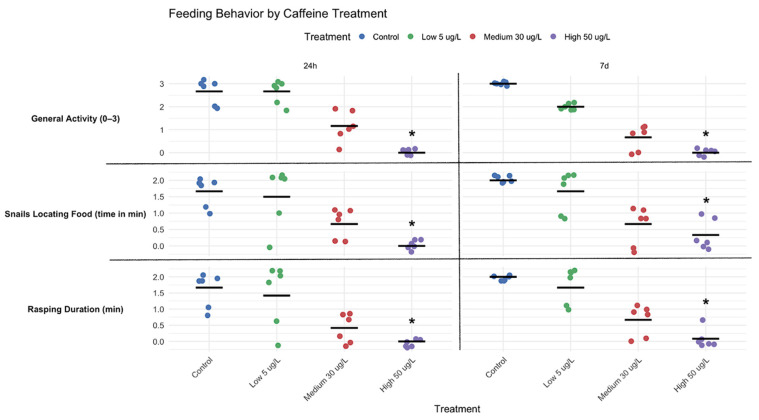
Feeding Behavior of *P. acuta* in Response to CAF Exposure. Significant differences in feeding behavior were observed between snails exposed to higher concentrations of CAF and those in the control group after 24 h. Significant differences from the control (*p* < 0.05) are indicated by asterisks. Data were visualized using RStudio (Posit Software, PBC, Version 2024.04.2+764).

The effect of CAF on *P. acuta* feeding rates was assessed under different concentrations. Statistical analysis indicated a significant decrease in the feeding rate of snails exposed to 50 µg/L CAF compared to the Control group at both the 24-h and 7-day timepoints (*p* < 0.05; Figure 6**.**). This effect was consistent across all three parameters assessed: the ability and duration to locate food, the duration of rasping, and overall activity in the presence of food (Figure 6). These findings may suggest a strong and persistent inhibitory effect of high CAF concentrations on feeding performance as well.

Although Dunn post hoc comparisons did not reveal statistically significant differences in feeding rate between the Control group and the Low (5 µg/L) or Medium (30 µg/L) treatments, a progressive decline was observed across the three parameters assessed: food location, rasping duration, and overall feeding activity particularly at the 7-day exposure period where a slight decrease is noted (Figure 6).

This trend suggests a dose-dependent suppression of feeding behavior, even at lower concentrations. These results align with the broader behavioral patterns observed in the snails (speed, exploration, and distance travelled), further reinforcing the hypothesis that CAF may disrupt ecologically relevant parameters in aquatic invertebrates.

### 3.3. Embryo Development and Cardiac Activity

Embryonic development of *P. acuta* was evaluated over 14 days under control, low, medium, and high CAF treatments, and cardiac activity was recorded throughout the experiment.

**Figure 7:**
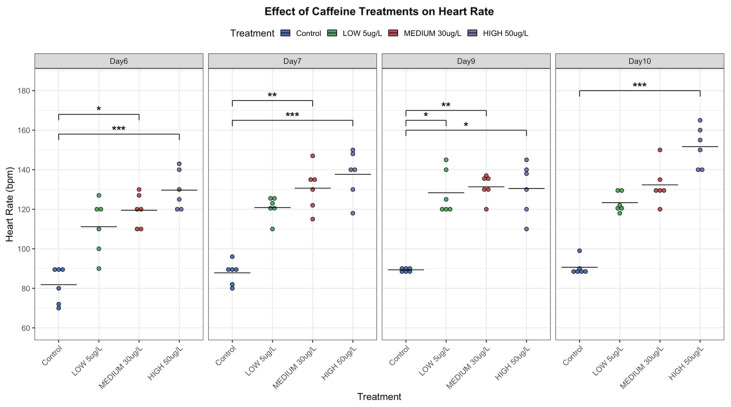
Heart Rate (BPM) of *P. acuta* following exposure to 5, 30, and 50 µg/L CAF. Significant Differences from Control Indicated by Asterisks (RStudio, Posit Software, PBC, Version 2024.04.2+764). * ≤0.05, ** ≤0.01 and *** ≤0.001. Note: Measurements began on Day 6 because the heart was fully developed at that point. Day 8 was excluded from the visualizations as its results were consistently similar to those of Day 7.

In relation to cardiac activity, the heart rate demonstrated a dose-dependent response. Specifically, low (5 µg/L) and medium (30 µg/L) concentrations of CAF led to a slight increase in the heart rate of snails (Control = 87 bpm, Low = 120 bpm, and Medium = 130 bpm). Although this increase was only statistically significant for 30 µg/L at day 6, 7 and 9, it suggests a general marginally accelerated activity compared to the control group (Figure 7). Additionally, minimal differences were observed between days.

The heart rates of snails exposed to elevated concentrations (50 µg/L) of CAF were significantly higher than those of the control group, indicating the potential of CAF to enhance cardiac activity (Figure 7). These results highlight CAF’s potential to enhance cardiac function in *P. acuta*.

In terms of embryonic development, embryos in the control and low-concentration groups achieved full development (score 5) by day 6, with a 100% hatching success rate. Low-concentration treatments (5 µg/L) exhibited comparable development, although some individuals displayed malformations in shell shape and radula; however, these effects were not statistically significant across all experiments.

**Figure 8:**
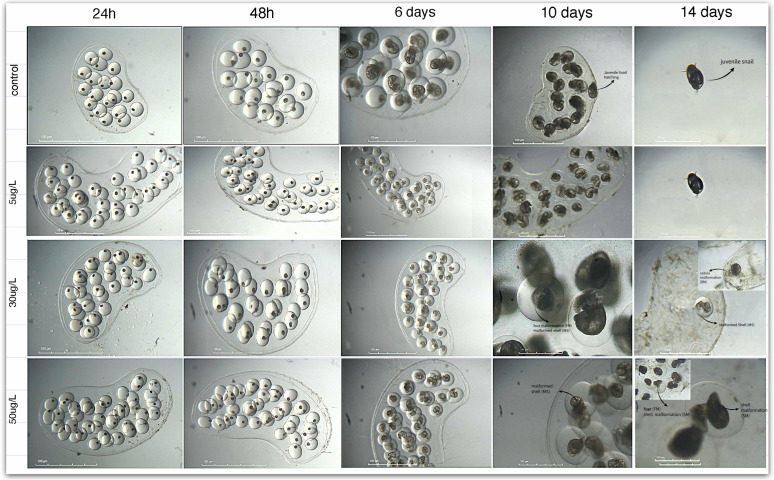
Effect of CAF (5, 30, and 50 µg/L) on the embryonic development of *P. acuta* from 24 h to juvenile hatching (day 14). At 50 µg/L CAF, no snail hatching was observed by day 14. At 30 µg/L, partial hatching occurred; however, some embryos exhibited malformations that interfered with the hatching process. Additional high-magnification images showing developmental progress and malformations at days 10 and 14 are included in the Supplementary Materials. Photographed Using Nikon SMZ-2T and MotiConnect Imaging System. Note: Further details on malformations by treatment and timepoint are provided in the Supplementary Material (Figures S1–S3).

At medium concentration, there were minor developmental delays and consistent malformations observed in approximately 2.5% of the specimens, while others developed completely, achieving a score of 4. Despite the presence of malformations, such as shell irregularities, and foot and radula malformations (Figure 8; Figures S1 and S2 Supplementary Material), the hatching rates at this concentration varied between 77% and 93% across the three experiments, with an average value of 86% at day 14 (Table 3). High concentration markedly impaired development, with replicates showing severe malformations (~60%) from day 7 onwards (Figure 8; Figures S1 and S3 Supplementary Material), resulting in a hatching success rate averaging 53.3% at day 14 (Table 3). These results demonstrate a clear dose-dependent inhibition of embryonic development, with significant toxicity observed at the highest concentration.

## 4. Discussion

This study offers new insights into the sublethal effects of caffeine (CAF) on freshwater gastropods, using *P. acuta* as a model species in ecotoxicological assessment. A range of ecologically relevant endpoints (locomotion, feeding rate, development, and heart rate) was examined to capture both behavioural and physiological responses. Results showed that environmentally relevant concentrations of CAF impact both behavioral and physiological responses in *P. acuta*, with effects differing based on concentration, exposure duration, and developmental stages.

During the embryonic phase, CAF induced heart rate changes dependent on both dosage and exposure time (Figure 7). Higher CAF concentrations (50 µg/L) increased heart rate, suggesting physiological stress or direct stimulation of the cardiac function. This dose also caused delayed development, morphological abnormalities, and decreased hatching success (Table 3), underscoring CAF’s potential to interfere with normal embryogenesis (Figure 8, Figure S3 Supplementary material). Similar developmental impairments were reported in other species: *Paracentrotus lividus* exhibited a reduction in embryo-larval development from 63% to 29%, as reported by [30], while zebrafish embryos exposed to CAF showed reduced body length and abnormal muscle fiber formation [10,31], and in *Chironomus riparius*, CAF altered ATP production, the primary energy source for cells, impairing larval growth and development [1].

In this context, the abnormalities and heart rate changes in *P. acuta* embryos suggest a dual effect of CAF, possibly tied to stage-specific organ development. Early exposure may disrupt cells formation (such as shell and radula alterations, see Figure 8, Figure S3 Supplementary Material), possibly due to altered ATP production [1]. While later effects may involve interference with nervous system formation. In freshwater gastropods, cardiac function is controlled by complex neural networks using multiple neurotransmitters (cholinergic, serotonergic, catecholaminergic) [32]. Thus, CAF may cause neurogenic disturbances during critical developmental windows that may play a critical role in the regulation of heart rhythm. This hypothesis, while preliminary, highlights the need for further research into stage-specific mechanisms of CAF toxicity. These findings suggest that embryonic exposure to CAF could have lasting consequences on *P. acuta*, potentially reducing survival and impacting freshwater trophic chains.

Adults *P. acuta* showed altered behavioral responses with short-term exposure (24 h) to low and medium concentrations of CAF (5 and 30 µg/L, respectively), increasing locomotor activity (Figure 3, Figure 4 and Figure 5), possibly due to CAF’s action as an adenosine receptor antagonist, increasing neural activity, movement, and reducing fatigue perception [17]. Additionally, it temporarily triggers increased exploratory behaviour, possibly as an alertness-driven response. This response aligns with CAF’s well-documented stimulant effects across both vertebrate and invertebrate taxa [17]. These behavioral alterations may be mediated by neurotransmitters such as acetylcholine, dopamine, or histamine [17]. Supporting this, studies on *Girardia tigrina* suggest these neuromodulators contribute to CAF-induced stimulation, with similar mechanisms described in dipterans and ecdysozoans [33].

However, it is important to note that this stimulatory effect was not observed at the highest concentration tested (50 µg/L). Instead, exposure to this concentration resulted in a significant decline in activity (Figure 3, Figure 4 and Figure 5). This may be indicative of physiological fatigue, stress adaptation, overstimulation, or the cumulative toxic effects associated with acute high-dose CAF exposure.

Interestingly, the results suggest a U-shaped (hormetic) dose–response pattern, where low and medium concentrations initially stimulate behavior, while higher concentrations are suppressive (Figure 3, Figure 4 and Figure 6). This phenomenon is consistent with findings regarding different chemicals in other taxa. In zoeal mud crabs, low concentrations of petroleum hydrocarbons increased megalopal weight, an effect that reversed at higher concentrations [34]. Similarly, low doses of cadmium enhanced reproductive output in snails, while higher doses proved lethal [35]. Hormesis is increasingly recognized in ecotoxicology as a complex and often overlooked response, particularly at sub-lethal exposure levels [36].

Interestingly, at lower concentrations, the initial stimulatory effects of CAF were not maintained over time. After seven days, individuals exposed to low and medium concentrations exhibited reduced movement, exploration, and feeding activity, almost comparable to those of control individuals (Figure 3, Figure 4 and Figure 5). This return to baseline may reflect physiological habituation to CAF, metabolic adaptation, or desensitization of neurotransmitter systems. Chronic exposures to stimulants, such as CAF, have been associated with adaptive responses such as receptor downregulation, energy reserves rebalancing, or modulation of oxidative stress pathways, which could normalize behavioural outputs over time [15]. These findings support the broader assumption in invertebrate research that CAF can diffuse into body tissues, including the central nervous system, allowing it to interact directly with neural pathways and modulate neurotransmitter activity over time [17].

These biphasic responses may complicate ecological risk assessments, as low-dose stimulation may appear harmless while masking adverse effects at higher or prolonged exposures. This pattern was evident in *P. acuta*, where short-term behavioural stimulation by CAF diminished with more prolonged exposure. These results highlight the need to account for both dosage and duration when assessing emerging contaminants such as CAF.

Meanwhile, CAF caused a dose-dependent decrease in snails’ feeding rate (Figure 6), likely due to its impact on dopaminergic pathways, which influence reduced feeding and aversion responses across multiple taxa, including *C. elegans* [37] and mammals [38]. A similar mechanism may operate in freshwater gastropods as *Lymnaea* [39], suggesting that CAF could alter *P. acuta*’s feeding patterns through neurochemical disruptions. However, further research is required.

Alterations in locomotion and feeding in *P. acuta* suggest interconnected neurobehavioral effects. Increased activity at lower doses may indicate stimulant-induced arousal or foraging. However, this was not accompanied by an increase in feeding, suggesting a decoupling of activity and appetite, perhaps attributable to differential effects on motor and dopaminergic pathways. Meanwhile, at higher doses or prolonged exposures, reduced feeding and movement (Figure 3, Figure 4, Figure 5 and Figure 6) indicate potential energy imbalance or neurotoxicity. Ecologically, these behaviour impairments can compromise energy acquisition and growth [40], snails’ ability to cope with environmental stressors or threats, predator avoidance, and habitat exploration, ultimately reducing population viability and ecological fitness. Such disruptions may lead to shifts in community structures and ecosystem dynamics.

Nevertheless, the molecular pathways underlying these effects in freshwater gastropods remain poorly understood [17], limiting interpretation, highlighting a key gap in ecotoxicological research. Addressing this is crucial, given the variability in responses observed across developmental stages. While adult snails exhibited reduced activity under high CAF exposure, embryos demonstrated increased heart rates and heightened sensitivity, including morphological abnormalities. These contrasting responses within the same species highlight the complexity of pollutant impacts and emphasize the necessity for multi-level, life-stage-inclusive assessments to more accurately evaluate ecological risks.

Notably, adverse effects seen in embryos of *P. acuta* are crucial for understanding how CAF pollution can disrupt food chains and compromise ecosystem stability. Addressing this is especially important considering that CAF is one of the most widely consumed psychoactive substances worldwide, due to its extensive use and incomplete removal during wastewater treatment. Additionally, heart rate measures in this study emerge as a promising, non-invasive physiological biomarker of sub-lethal stress responses in embryos of freshwater invertebrates. *P. acuta* proves to be a valuable model for ecotoxicology, sensitive to pollutants, and exhibits clear behavioral and physiological responses, which makes it a practical model for assessing the impacts of emerging contaminants such as pharmaceuticals and personal care products in freshwater environments.

## 5. Conclusions

CAF, a widely consumed and increasingly detected environmental contaminant, poses potential ecotoxicological risks to aquatic ecosystems [1,16]. Using *P. acuta* as a model organism, it was found that environmentally relevant concentrations of CAF can significantly alter behavioural patterns, feeding rates, embryo heart rate, and development. Sublethal effects, particularly 50 µg/L, suggest that CAF may affect multiple life stages of key species and weaken community resilience. Importantly, this research highlights the value of specific bioassays (e.g., feeding rates and embryonic heart rate measures), offering a non-invasive and cost-effective means of detecting ecologically relevant effects. However, limited understanding of the molecular and cellular mechanisms involved, especially in freshwater gastropods, hinders interpretation of the ecological outcomes [17].

As CAF is almost recognized as a contaminant of emerging concern [29,41,42], future research should adopt a multidisciplinary approach and consider how environmental variables, chemical mixtures, and climate stressors may influence its toxicity to better predict its long-term ecological impact.

## Figures and Tables

**Table 1:** Embryonic growth, stages of development and characteristic features in *P. acuta*. * Basommatophora: freshwater mollusks, like *Physella* spp., have the eyes located next to the base of the tentacles.

Stage Name	Description	Approx. Time After Oviposition
**Fertilized Egg**	Zygotes encased in a gelatinous egg capsule	0 h
**Cleavage**	Rapid mitotic divisions form a blastula (multicellular sphere)	4–6 h
**Gastrula**	Embryo with germ layers (ectoderm, mesoderm, endoderm) begin to form	12–18 h
**Trochophore**	Beginning of organogenesis; “grainy” embryo, uninterrupted rotation, prototroch rises over body surface, embryo is still rounded	24–30 h
**Early Veliger**	Formation of shell (with helical torsion) and foot	3 days
**Late Veliger**	Development of eye spots (basomatophores *); radula; filiform and cylindrical antennas; and internal organs (heart and lung)	5–9 days
**Hatching**	Juvenile snail emerges from the egg capsule	12–14 days
**Juvenile**	Snail grows; full organ development; active feeding and movement	2 weeks
**Adult**	Reaches sexual maturity; capable of reproduction (hermaphroditic)	~2 months

**Table 2:** Developmental Scoring System for Embryonic Stages of *P. acuta*. Based on Morphological and Functional Features (0–5 Scale).

Score	Developmental Features	Description
**0**	Malformed or arrested	No visible development, disorganized structure, or major malformations
**1**	Early cleavage only	Embryo shows only early cleavage stages (2–16 cells), no organogenesis
**2**	Gastrula or early morphogenesis	Clear tissue layers forming; no visible organ structures yet
**3**	Shell formed, no organ structures	Embryo forms a recognizable body shape and shell, but lacks visible organs
**4**	Shell + organs (heart/eyes) visible	Partial organogenesis, shell and either heart or eyes present
**5**	Fully developed—shell, heart, and eyes present and functional	Advanced development with shell, heart, foot, and eyes well-formed and active (e.g., heartbeat)

**Table 3:** Percentage hatching mean values of replicates and experiments for each treatment group measured on day 14. The control and 5 µg/L treatments showed no reduction, while 30 µg/L and 50 µg/L treatments resulted in decreases to approximately 86% and 53% of the initial values, respectively.

Treatments	% Hatching Mean at Day 14
**Control**	100
**5 µg/L**	100
**30 µg/L**	86
**50 µg/L**	53

## Data Availability

The original contributions presented in this study are included in the article/supplementary material. Further inquiries can be directed to the corresponding author.

## Supplementary Materials

The following supporting information can be downloaded at: https://www.mdpi.com/article/10.3390/toxics14010014/s1, Figure S1: Effect of Caffeine (5, 30, and 50 µg/L) on the embryonic development of *Physa acuta* from 10 days to juvenile hatching (day 14); Figure S2: Effect of medium concentration of Caffeine (30 µg/L) on embryonic malformation seen in *Physa acuta* from day 7 to 14; Figure S3: Effect of High Concentration of Caffeine (50µg/L) on Embryonic Malformation seen in *Physa acuta* from Day 7 to 14; Table S1: Summary of malformations observed in *Physella acuta* embryos exposed to Caffeine.

## Abbreviations

The following abbreviations are used in this manuscript:

CAF	Caffeine
NMR	Nuclear magnetic resonance

## References

[1] Rodrigues S., Alves R.S., Antunes S.C. (2025). Impact of Caffeine on Aquatic Ecosystems: Assessing Trophic-Level Biological Responses. J. Xenobiotics.

[2] Bruton T., Alboloushi A., De La Garza B., Kim B.-O., Halden R.U. (2010). Fate of caffeine in the environment and ecotoxicological considerations. Contaminants of Emerging Concern in the Environment: Ecological and Human Health Considerations.

[3] Razali S.M., Masimen M.A.A., Husain N., Idris I., Ismail W.I.W., Hamid H.A. (2025). Global Trends in Caffeine-based Lifestyles: A CiteSpace Exploration of Potential Environmental Sustainability Impacts. Int. J. Pharm. Nutraceuticals Cosmet. Sci..

[4] Wada O.Z., Olawade D.B. (2025). Recent occurrence of pharmaceuticals in freshwater, emerging treatment technologies, and future considerations: A review. Chemosphere.

[5] Koral M., Ergül H. (2024). Assessment of Caffeine and its metabolites on marine phytoplankton growth. Res. Mar. Sci..

[6] Korekar G., Kumar A., Ugale C. (2020). Occurrence, fate, persistence and remediation of caffeine: A review. Environ. Sci. Pollut. Res..

[7] de Cravalho A.C.C., da Silva Paganini W., de Almeida Piai K., Bocchiglieri M.M. (2024). The presence of pharmaceuticals and caffeine in water, as well as the methods used to eliminate them. Curr. Opin. Environ. Sci. Health.

[8] Pashaei R., Dzingelevičienė R., Putna-Nimane I., Overlinge D., Błaszczyk A., Walker T.R. (2023). Acute toxicity of triclosan, caffeine, nanoplastics, microplastics, and their mixtures on *Daphnia magna*. Mar. Pollut. Bull..

[9] Lopez C., Nnorom M.-A., Tsang Y.F., Knapp C.W. (2021). Pharmaceuticals and personal care products’(PPCPs) impact on enriched nitrifying cultures. Environ. Sci. Pollut. Res..

[10] Diogo B.S., Antunes S.C., Pinto I., Amorim J., Teixeira C., Teles L.O., Golovko O., Žlábek V., Carvalho A.P., Rodrigues S. (2023). Insights into environmental caffeine contamination in ecotoxicological biomarkers and potential health effects of *Danio rerio*. Heliyon.

[11] Ferreira A.P. (2005). Caffeine as an environmental indicator for assessing urban aquatic ecosystems. Cad. Saúde Pública.

[12] Vieira L., Soares A., Freitas R. (2022). Caffeine as a contaminant of concern: A review on concentrations and impacts in marine coastal systems. Chemosphere.

[13] Quadra G.R., Paranaíba J.R., Vilas-Boas J., Roland F., Amado A.M., Barros N., Dias R.J.P., Cardoso S.J. (2020). A global trend of caffeine consumption over time and related-environmental impacts. Environ. Pollut..

[14] Edwards Q.A., Kulikov S.M., Garner-O’Neale L.D. (2015). Caffeine in surface and wastewaters in Barbados, West Indies. SpringerPlus.

[15] Nunes B., Santos J., Dionísio R., Dias de Alkimin G. (2022). Investigation of potential behavioral and physiological effects of caffeine on D. magna. Environ. Sci. Pollut. Res..

[16] Li S., Wen J., He B., Wang J., Hu X., Liu J. (2020). Occurrence of caffeine in the freshwater environment: Implications for ecopharmacovigilance. Environ. Pollut..

[17] Mustard J.A. (2014). The buzz on caffeine in invertebrates: Effects on behavior and molecular mechanisms. Cell. Mol. Life Sci..

[18] Raynal R.S., Schwanz L.E., Bonduriansky R. (2025). Differential effects of ambient temperature on juvenile versus adult life-stages of an invasive freshwater snail. Evol. Ecol..

[19] Sereshti H., Samadi S. (2014). A rapid and simple determination of caffeine in teas, coffees and eight beverages. Food Chem..

[20] Al-Maaieh A., Flanagan D.R. (2002). Salt effects on caffeine solubility, distribution, and self-association. J. Pharm. Sci..

[21] Sánchez-Argüello P., Fernández C., Tarazona J.V. (2009). Assessing the effects of fluoxetine on *Physa acuta* (Gastropoda, Pulmonata) and *Chironomus riparius* (Insecta, Diptera) using a two-species water–sediment test. Sci. Total Environ..

[22] Martínez-Paz P., Morales M., Sánchez-Argüello P., Morcillo G., Martínez-Guitarte J.L. (2017). Cadmium in vivo exposure alters stress response and endocrine-related genes in the freshwater snail *Physa acuta*. New biomarker genes in a new model organism. Environ. Pollut..

[23] Prieto-Amador M., Caballero P., Martinez-Guitarte J.-L. (2021). Analysis of the impact of three phthalates on the freshwater gastropod *Physella acuta* at the transcriptional level. Sci. Rep..

[24] Chiara V., Kim S.Y. (2023). AnimalTA: A highly flexible and easy-to-use program for tracking and analysing animal movement in different environments. Methods Ecol. Evol..

[25] Fodor I., Schmidt J., Svigruha R., László Z., Molnár L., Gonda S., Elekes K., Pirger Z. (2025). Chronic tributyltin exposure induces metabolic disruption in an invertebrate model animal, *Lymnaea stagnalis*. Aquat. Toxicol..

[26] Marchant D.J., Perkins D.M., Jones J.I., Kratina P. (2025). Physiological and behavioural responses of aquatic organisms to microplastics and experimental warming. Environ. Pollut..

[27] Hollingsworth R.G., Armstrong J.W., Campbell E. (2003). Caffeine as a novel toxicant for slugs and snails. Ann. Appl. Biol..

[28] Chemin E. (1926). Les Mollusques d’eau Douce.

[29] Moore M., Greenway S., Farris J., Guerra B. (2008). Assessing caffeine as an emerging environmental concern using conventional approaches. Arch. Environ. Contam. Toxicol..

[30] Li S., He B., Wang J., Liu J., Hu X. (2020). Risks of caffeine residues in the environment: Necessity for a targeted ecopharmacovigilance program. Chemosphere.

[31] Chen Y.-H., Huang Y.-H., Wen C.-C., Wang Y.-H., Chen W.-L., Chen L.-C., Tsay H.-J. (2008). Movement disorder and neuromuscular change in zebrafish embryos after exposure to caffeine. Neurotoxicol. Teratol..

[32] Kodirov S.A. (2011). The neuronal control of cardiac functions in Molluscs. Comp. Biochem. Physiol. Part A Mol. Integr. Physiol..

[33] Omond S.E., Hale M.W., Lesku J.A. (2022). Neurotransmitters of sleep and wakefulness in flatworms. Sleep.

[34] Agathokleous E., Barceló D., Fatta-Kassinos D., Moore M.N., Calabrese E.J. (2021). Contaminants of emerging concern and aquatic organisms: The need to consider hormetic responses in effect evaluations. Water Emerg. Contam. Nanoplastics.

[35] Jiang H., Zhang Y., Zhang L., Mao L., Zhao Z., Sial M.U. (2024). Studying the phenomenon of hormesis and its effect on insects. Entomol. Appl. Sci. Lett..

[36] Sebastiano M., Messina S., Marasco V., Costantini D. (2022). Hormesis in ecotoxicological studies: A critical evolutionary perspective. Curr. Opin. Toxicol..

[37] Min H., Youn E., Kawasaki I., Shim Y.-H. (2017). Caffeine-induced food-avoidance behavior is mediated by neuroendocrine signals in *Caenorhabditis elegans*. BMB Rep..

[38] Correa M., SanMiguel N., López-Cruz L., Carratalá-Ros C., Olivares-García R., Salamone J.D. (2018). Caffeine modulates food intake depending on the context that gives access to food: Comparison with dopamine depletion. Front. Psychiatry.

[39] Aonuma H., Kaneda M., Hatakeyama D., Watanabe T., Lukowiak K., Ito E. (2016). Relationship between the grades of a learned aversive-feeding response and the dopamine contents in *Lymnaea*. Biol. Open.

[40] Sokolova I.M., Frederich M., Bagwe R., Lannig G., Sukhotin A.A. (2012). Energy homeostasis as an integrative tool for assessing limits of environmental stress tolerance in aquatic invertebrates. Mar. Environ. Res..

[41] Picinini-Zambelli J., Garcia A.L.H., Da Silva J. (2025). Emerging pollutants in the aquatic environments: A review of genotoxic impacts. Mutat. Res. Rev. Mutat. Res..

[42] Yang Y., Wan Y., Chen H., Li Y., Muñoz-Carpena R., Zheng Y., Huang J., Zhang Y., Gao B. (2025). Caffeine removal in wastewater: A comprehensive review of current treatment plants and small-scale innovations. Environ. Technol. Rev..

