# Supplementary material for: Effects of Caffeine Exposure on Behaviour, Development and Physiology of the Freshwater Snail Physella acuta (Draparnaud, 1805)
Source: Toxics. 2025 Dec 22;14(1):14. doi: 10.3390/toxics14010014 (PMC12846127; doi:10.3390/toxics14010014)
Supplement: Supplementary file 1 [file toxics-14-00014-s001.zip › toxics-4032858-supplementary.pdf]

SUPPLEMENTARY MATERIAL

Effects of Caffeine Exposure on Behaviour, Development and Physiology of the Freshwater Snail *Physella acuta* (Draparnaud, 1805)

Ahlam Mohamed-Benhamu

Grupo de Bioinformática y Ecotoxicología Molecular de Invertebrados, Facultad de Ciencias, Universidad Nacional de Educación a Distancia (UNED), Av. Esparta S/N, 28232 Madrid, Spain; ahlam.mohamed@ccia.uned.es; Tel.: +34-91-398-7644

**Physella acuta=Physa acuta*

Table S1 Summary of malformations observed in *Physella acuta* embryos exposed to Caffeine. Exposure to 30 and 50 µg/L caffeine resulted in a range of developmental abnormalities. At 30 µg/L, partial hatching was observed, but several embryos displayed morphological defects including shell malformations [irregular or cup-shaped shells lacking coiling and foot deformities (Figure S2)]. At 50 µg/L, no hatching occurred by day 14, and embryos exhibited severe disruptions such as delayed organogenesis, radula malformations and shortened antennae (Figure S3). These malformations interfered with normal development and hatching. Images from days 10 and 14 are provided in the supplementary materials. Images captured using Nikon SMZ-2T stereomicroscope and A1 MotiConnect Imaging System

Malformation or Development Problem Type	Abbreviation	Description	CAF Concentration (µg/L)	Developmental Stage (Day)
Shell Malformation (General)	**SM**	Irregular, incomplete, or asymmetric shell formation	30, 50	Day 10 and 14
Shell Malformation (Cup-Shaped)	**SM**	Cup-shaped shell lacking normal coiling	30, 50	Day 14
Antenna Malformation	**AM**	Abnormally short, or absent tentacles	30, 50	Day 10 and 14
Radula Malformation	**RM**	Malformed or missing radula, affecting feeding ability	30,50	Day 7, 10 and 14
Foot Malformation	**FM**	Underdeveloped foot, impairing movement or hatching	30, 50	Day 10 and 14
Incomplete Hatching	**-**	Embryo fully developed but unable to emerge from the egg capsule	50	Day 14

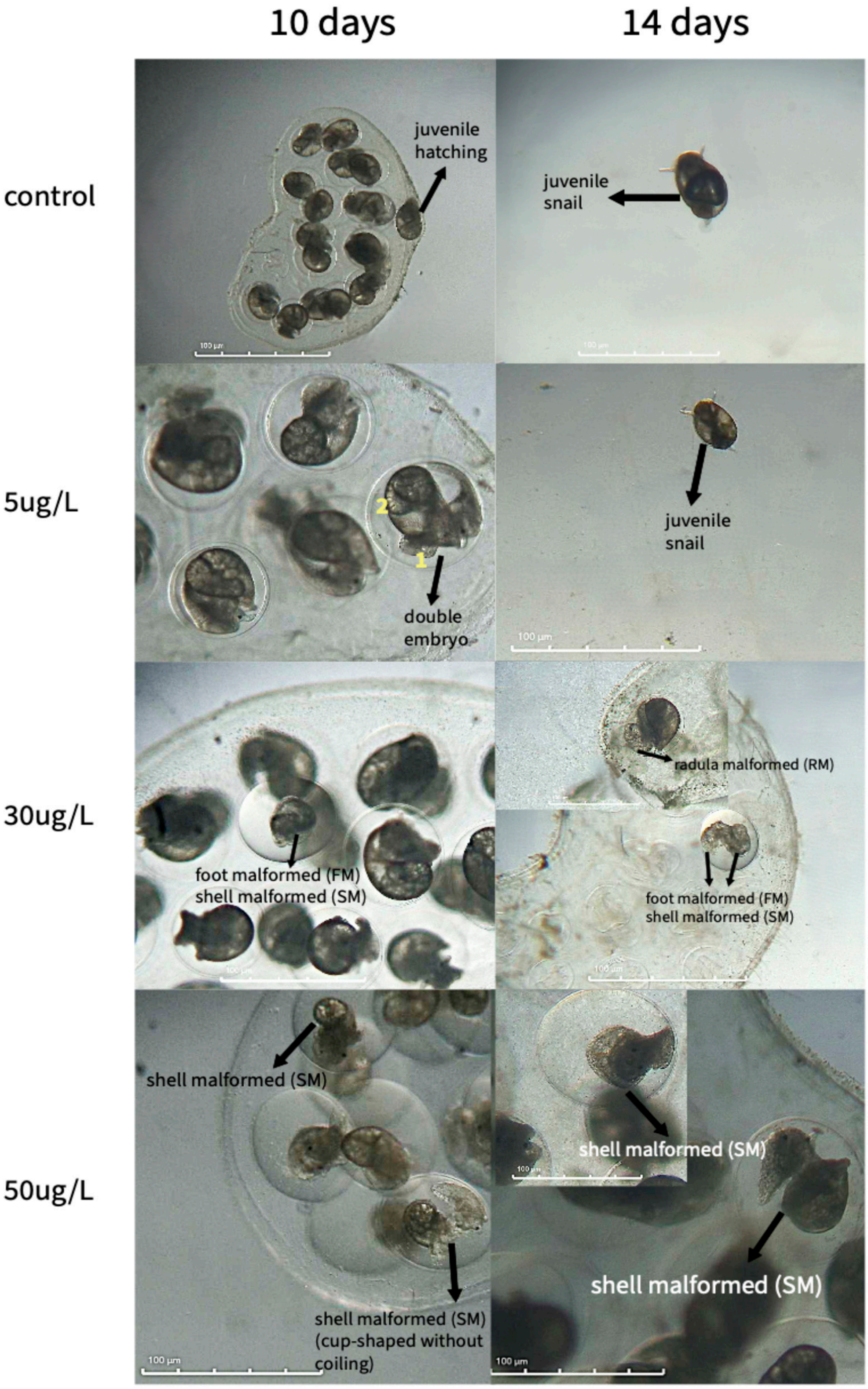

Figure S1. Effect of Caffeine (5, 30, and 50 µg/L) on the embryonic development of *Physa acuta* from 10 days to juvenile hatching (day 14). At 50 µg/L caffeine, no snail hatching was observed by day 14. At 30 µg/L, partial hatching occurred; however, some embryos exhibited malformations that interfered with the hatching process. An overall image is provided in the main manuscript. All images were captured using a Nikon SMZ-2T stereomicroscope and the A1 MotiConnect Imaging System.

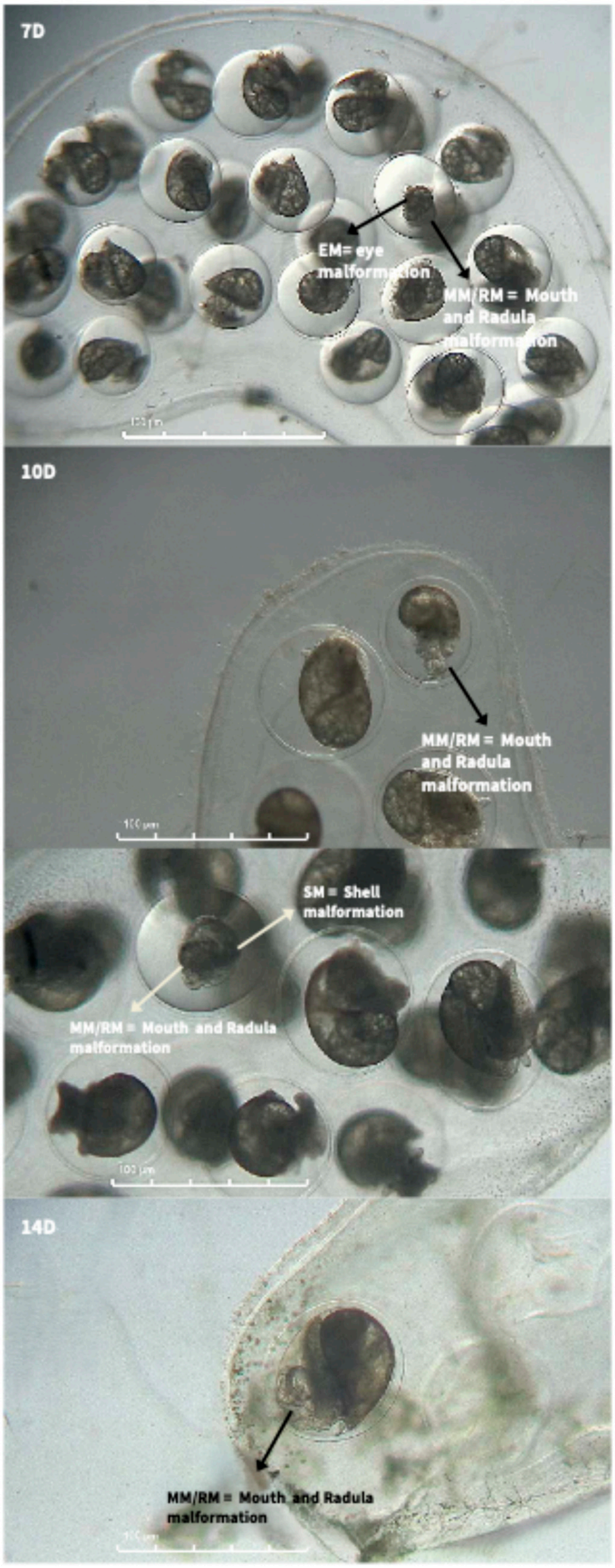

Figure S2. Effect of medium concentration of Caffeine (30 µg/L) on embryonic malformation seen in *Physa acuta* from day 7 to 14. At 30 µg/L, partial hatching occurred; however, some embryos exhibited malformations that interfered with the hatching process. Photographed Using Nikon SMZ-2T and A1 MotiConnect Imaging System

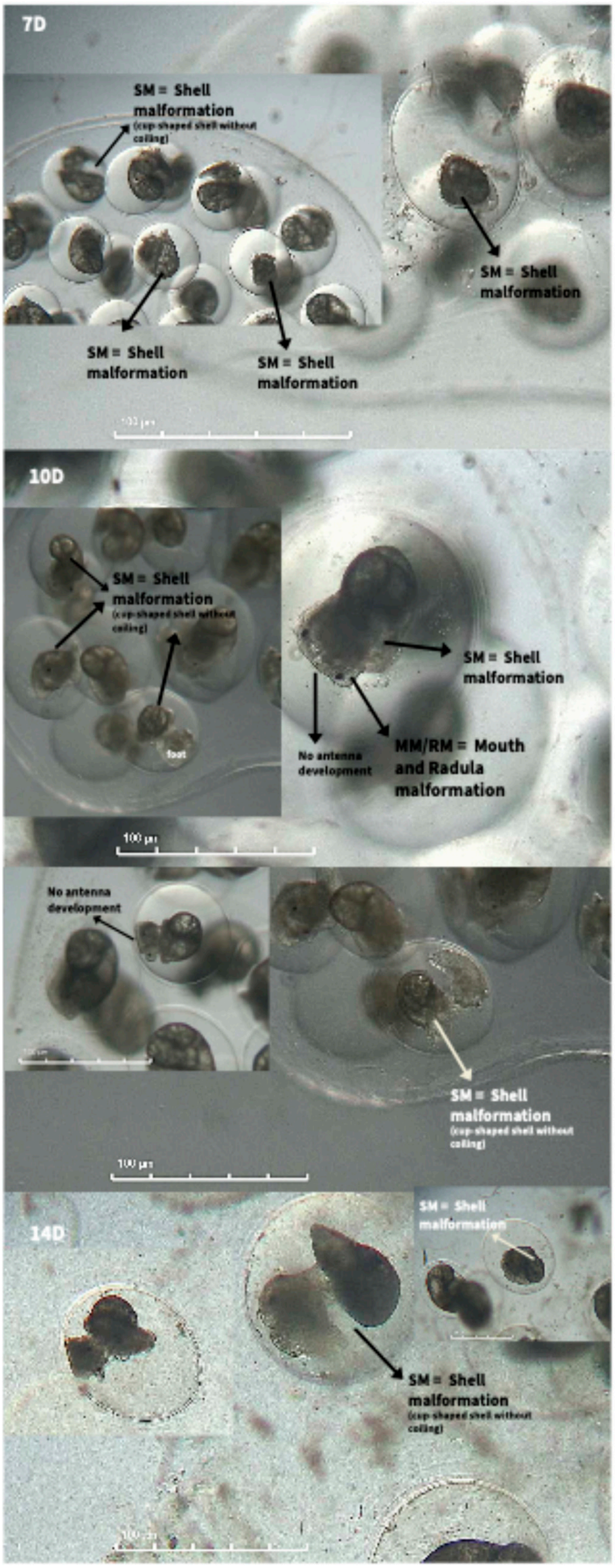

Figure S3. Effect of High Concentration of Caffeine (50µg/L) on Embryonic Malformation seen in *Physa acuta* from Day 7 to 14. At 50 µg/L, no hatching occurred by day 14, and embryos exhibited severe disruptions such as delayed organogenesis, radula malformations and shortened antennae Photographed Using Nikon SMZ-2T and A1 MotiConnect Imaging System
